# Morphological, Morphometrical and Molecular Characterization of *Oscheius siddiqii* Tabassum and Shahina, 2010 (Rhabditida, Rhabditidae) from India with Its Taxonomic Consequences for the Subgenus *Oscheius* Andrássy, 1976

**DOI:** 10.3390/biology10121239

**Published:** 2021-11-27

**Authors:** Aashaq Hussain Bhat, Swati Gautum, Aasha Rana, Ashok Kumar Chaubey, Joaquín Abolafia, Vladimír Půža

**Affiliations:** 1 Department of Zoology, Government Degree College Uttersoo, Anantnag 192201, India; aashiqhussainbhat10@gmail.com; 2 Experimental Biology Research Group, Institute of Biology, Faculty of Science, University of Neuchatel, Rue Emile-Argand 11, 2000 Neuchatel, Switzerland; 3 Nematology Laboratory, Department of Zoology, Chaudhary Charan Singh University, Meerut 250004, India; swatigautamswati22@gmail.com (S.G.); aasha.aasharana@ymail.com (A.R.); akc.nema@gmail.com (A.K.C.); 4 Departamento de Biología Animal, Biología Vegetal y Ecología, Universidad de Jaén, Avenida de Ben Saprut s/n, 23071 Jaén, Spain; abolafia@ujaen.es; 5 Biology Centre, Institute of Entomology, Czech Academy of Sciences, Branišovská 31, 370 05 České Budějovice, Czech Republic

**Keywords:** *Oscheius*, systematics, phylogenetic analysis

## Abstract

**Simple Summary:**

Due to their potential entomopathogenicity, nematodes of the genus *Oscheius* have been in the spotlight of the scientific interest in recent years. Unfortunately, some of these species are poorly described and have inadequate or no molecular support. This fact complicates the systematics of the group, and a revision of these species is necessary to elucidate their taxonomic status. In the present study, we provide a detailed description based on the morphological, morphometrical, and molecular characteristics of *Oscheius siddiqii* from Uttar Pradesh, India, including the first scanning electron microscopy (SEM) studies of the species. Furthermore, based on morphological and molecular data, the status of some *Oscheius* species is discussed, and several synonymisations are proposed.

**Abstract:**

An insect parasitic nematode belonging to the genus *Oscheius* was recovered from the agricultural soils from the Hapur district in western Uttar Pradesh, India. Morphological studies on this species exhibited its high resemblance with two Pakistani species: *Oscheius* *siddiqii* and *O. niazii*. No molecular data are available for these taxa but, morphologically, both species do not differ significantly from our strains and each other. Hence, these nematodes can be considered conspecific, and the correct name for this taxon is *O. siddiqii*, the first described species. The phylogenetic analyses of the ITS-, 18S-, and the 28S rDNA sequences showed that *O. siddiqii* is a sister taxon to the group formed by *Oscheius* *microvilli*, *O. myriophilus,* *O. safricanus*, and several unidentified *Oscheius* species. Additionally, our analyses show that based on molecular and morphological data, the species *Oscheius rugaoensis* and *O. microvilli* cannot be distinguished from *O. chongmingensis* and *O. myriophilus*, respectively, and are thus considered junior synonyms of these taxa. Furthermore, the available data are not sufficient to evaluate the status of *Oscheius basothovii* and *O. safricanus,* which are, in consequence, considered *species inquirendae*. These findings highlight the necessity of the proper morphological and molecular characterisation of the described *Oscheius* species.

## 1. Introduction

The genus *Oscheius* was proposed by Andrássy [1], with *O. insectivorus* (=*Rhabditis insectivora* Körner in Osche 1952) as type species. Sudhaus [2,3] distinguished two groups into the genus *Oscheius*, the *Insectivorus*-group, which includes species with a bursa leptoderan or pseudopeloderan and spicules with a crochet needle-shaped tip, and the *Dolichura*-group, which includes species with a peloderan bursa and spicules with a thin tubular tip. More recently, Abolafia and Peña-Santiago [4] categorised both the groups as subgenera, dividing them into *Oscheius*: *Oscheius* and *Dolichorhabditis*, respectively, according to the division proposed by Andrássy [5,6]. With respect to the subgenus *Oscheius* (or insectivorus-group), 23 valid species have been described at the time of writing [4,7] (Supplementary Table S1).

Unfortunately, some of these species are poorly described and have inadequate or no molecular support. This fact complicates the systematics of the group, and a revision of these species is necessary to elucidate their taxonomic status. In our recent study [7], we compared an Indian population of *Oscheius* sp. from India and six *Oscheius* species described from Pakistan (*Oscheius citri, O. cobbi, O. cynodonti, O. esculentus, O. punctatus,* and *O. sacchari*). The morphological comparison and molecular analysis of the only available genetic marker for the Pakistani species, the ITS rDNA sequence, showed that these nematodes do not differ from our Indian population and from each other; hence, they can be considered synonyms, and the correct name for this taxon is the first described species, *O. citri*. Such kinds of studies can significantly clarify group systematics and can improve our understanding of species diversity.

The molecular taxonomy of nematodes relies on phylogenetic reconstructions that are based on three nuclear ribosomal DNA genetic markers (conserved and variable regions of the 18S and 28S subunits and the more variable ITS region) [8]. In some nematode groups, the 18S and 28S genes lack the resolution that is required to distinguish between these closely-related lineages [9]. The quickly evolving internal transcribed spacer (ITS) region may display intrapopulation, or even individual variability [10,11], which could complicate its use in systematics [12]. Mitochondrial genes thus represent a viable option in nematode systematics [12]. Unfortunately, so far, no mitochondrial gene sequences are available for *Oscheius* nematodes, with the only exception being *Oscheius onirici,* which has several published sequences of the cytochrome oxidase I (COI) gene available in the NCBI GenBank database [13,14].

During the course of the nematological sampling survey in the Hapur district of western Uttar Pradesh, India, two *Oscheius* populations, labelled CS42 and CS43, were isolated from agricultural soil samples using the *Galleria* trap method [15] and were later morphologically identified as *O. siddiqii* Tabassum & Shahina, 2010.

*Oscheius siddiqii* was first described from soil samples taken from around the roots of rose (*Rosa damascena* L.) in Karachi, Sindh, Pakistan [16]. This species was previously described based on morphological and morphometric studies alone. In the present study, a detailed description based on the morphological, morphometrical, and molecular (small subunit, large subunit, and internal transcribed spacer (ITS1-5.8S-ITS2) rDNA gene sequences) characteristics of *O. siddiqii* from Uttar Pradesh, India, is provided, including the first scanning electron microscopy (SEM) studies of the species. We also provide the first COI sequences of *O. siddiqii* and several others *Oscheius* species. Furthermore, based on morphological and molecular data, the status of some *Oscheius* species is discussed.

## 2. Materials and Methods

### 2.1. Nematode Source

Twenty soil samples were collected in different agricultural fields of the Brijghat area of the Hapur district (28°47′ N, 78°4′ E and elevation of 219 m above sea level) in western Uttar Pradesh, India, in February 2018. The predominant climate in these areas is semi-arid and moderate to tropical monsoon (humid subtropical). Each sample had 1.5 kg of soil, which was made up of five soil subsamples collected at five sites within each field (one sample from each corner of the field and one from the centre of the field). Samples were collected at a 15–20 cm depth around the rhizosphere of crop plants [17]. These samples were tested for the presence of nematodes using the *Galleria* soil baiting technique [15]. Two soil samples taken from sugarcane fields (*Saccharum officinarum* L.) and cucumber (*Cucumis sativus* L.) cultivars were found to be positive for the presence of nematodes and were labelled as CS42 and CS43. The pH of the soil samples ranged from 9.6–10.0. The cadavers of *Galleria mellonella* L. (Lepidoptera, Pyralidae) from the soil baits were transferred to white traps after proper washing with double distilled water and sterilization with 0.1% NaOCl [12]. The nematodes that emerged from the white trap within 6–10 days were collected. The nematode cultures were kept in 250 mL flasks in the Bio-Oxygen Demand incubators at a temperature of 15 °C.

Slides and live specimens are also deposited at CCS University Meerut, University of Jaén, and the Institute of Entomology Czech Republic, respectively. Scanning electron microscopy and light microscopy images were taken at the University of Jaén, while the molecular studies were conducted at CCS University Meerut and at the Institute of Entomology, Czech Republic.

### 2.2. Morphological and Morphometrical Characterization

Prior to the light microscopic studies and morphometry analyses, the nematodes were propagated using the last instar larvae of *G. mellonella* by injecting 10 greater wax moth larvae with approximately 2000 surface-sterilized dauer larvae in sterilized Petri dishes using a 1 mL insulin syringe. The larvae died within 48–72 h [7]. Adult generations (females and males) and 3rd stage juveniles were isolated from the white trap within 7–10 days [18]. These were killed with hot water at 60 °C [19], fixed in TAF (7 mL formalin, 2 mL triethanolamine, 91 mL ddH_2_O) [20], dehydrated by means of Seinhorst’s [21] method, and processed to glycerine [22]. The nematodes were mounted in a small drop of glycerine on permanent glass slides and were sealed with an extra amount of paraffin wax to prevent the flattening of the nematodes [23]. The observations and measurements of 136 specimens were taken using the Nikon DS-L1 image acquisition software mounted on a phase-contrast microscope (Nikon Eclipse 50i) in µm (Table 1 and Table 2). A Nikon Eclipse 80i (Nikon, Tokyo, Japan) light microscope with differential interference contrast optics and a Nikon Digital Sight DS-U1 camera were used to photograph the best-preserved nematode specimens. Adobe^®^ Photoshop^®^ CS was used to process the micrographs. Nematode species were identified based on morphological and morphometric features using the keys described by Abolafia and Peña-Santiago [4]. The morphological and morphometrical traits used to characterise the species are given in Table 1 and Table 2, and the morphometrical data were analysed in SPSS software to calculate different central tendency measures (Table 1).

### 2.3. Scanning Electron Microscopy

For the scanning electron microscopy, male and female specimens kept in glycerol were chosen for observation according to the protocol proposed by Abolafia [24]. The nematodes were hydrated in dH_2_O, dehydrated in a graded ethanol–acetone series, critical point dried with dry ice, mounted on SEM stubs with copper tape, and coated with gold in a sputter coater. Specimens were observed, and the microphotographs for ach sample were captured with a Zeiss Merlin microscope (5 kV) (Zeiss, Oberkochen, Germany). All of the light and scanning electron microscopy images were submitted to the X public database.

### 2.4. Molecular Analyses

DNA was isolated from the females and from the bulk of the dauer larvae (*n* > 500) (IJs). Individual females were placed in sterilized microcentrifuge tubes (250 µL) containing 10 µL of extraction buffer (8.85 µL of ddH_2_O, 1 µL of 10 × PCR buffer, 0.1 µL of 1% Tween, and 0.05 µL of proteinase K). Samples were frozen at –20 °C for 20 min and were then kept at 65 °C for 1 h and finally at 95 °C for 10 min. The lysates were cooled on ice and centrifuged (2 min, 9000 g), and 1 µL of supernatant was used for PCR [18]. The DNA from the pool of IJs was isolated using the Qiagen Blood and Tissue Analysis Kit (Hilden, Germany) according to the instructions of the manufacturer. For each locus that was amplified (see further), the DNA from five females and from the bulk of dauer larvae were used. A section of the rDNA comprising the internally transcribed spacer regions (ITS1-5.8S-ITS2) was amplified using the following 18S primers: 5′-TTGATTACGTCCCTGCCCTTT-3′ (forward) and 28S: 5′-TTTCACTCGCCGTTACTAAGG-3′ (reverse) [25]. The other section comprising the D2-D3 expansion domains of the 28S rDNA gene (large subunit, LSU) was amplified using the following D2F primers: 5′-CCTTAGTAACGGCGAGTGAAA-3′ (forward) and 536: 5′-CAGCTATCCTGAGGAAAC-3′ (reverse) [26]; a region comprising 18S rDNA (small subunit, SSU) was determined using the following 18S-F primer pairs: 5′-GATACCGCCCTAGTTCTGACC-3′ and 18S-R: 5′-ACCAACTAAGAACGGCCATG-3′ [27] and G18S4: 5′-GCTTGTCTCAAAGATTAAGCC-3′ and 18P: 5′-TGATCCWMCRGCAGGTTCAC-3′ [28].

The PCR master mix included 7.25 µL of ddH_2_O, 1.25 µL of 10 × PCR buffer, 1 µL of dNTPs, 0.75 µL of each forward and reverse primers, 0.1 µL of DNA polymerase, and 1 µL of DNA-extract. The PCR profiles were used as follows for ITS: 1 cycle of 94 °C for 7 min followed by 35 cycles of 94 °C for 60 s, 50 °C for 60 s, 72 °C for 60 s, and a final extension at 72 °C for 7 min [26]; for 28S rDNA: 1 cycle of 94 °C for 7 min followed by 35 cycles of 94 °C for 60 s, 55 °C for 60 s, 72 °C for 60 s, and a final extension at 72 °C for 10 min [29]; for 18S rDNA: 1 cycle of 94 °C for 5 min followed by 37 cycles of 94 °C for 60 s, 55 °C for 90 s, 72 °C for 2 min, and a final extension at 72 °C for 10 min [30]. After PCR, 2 µL of PCR product was electrophoresed in a 1% TAE-buffered agarose gel stained with ethidium bromide (20 µL ETB per 100 mL of gel) for 45 min at 120 V [31]. For each locus, PCR products from five females were sequenced by Eurofins Genomics (Ebersberg, Germany) and PCR-product from a bulk of dauer juveniles was sequenced by Bioserve PVT Ltd. (Hyderabad, India). No variation was detected in any of the products for each locus except for the ITS region of the strain CS42, and one sequence for each fragment was deposited in GenBank under accession numbers MH837093 (ITS sequence, strain CS43, the amplicon length 939 bp), MH819729 (28S sequence, CS42, amplicon length 885 bp), MT835468 (18S sequence, strain CS42, amplicon length 1571 bp), and MH845237 (28S sequence, CS43, amplicon length 885 bp). Due to the presence of variability in the ITS region of the strain CS42, an additional 15 individuals were sequenced for this marker. The two sequence variants were deposited under the accession numbers MH837095 and MT835467 (ITS sequences, strain CS42, and amplicon lengths 1010 bp and 990 bp).

### 2.5. Sequence Alignment and Phylogenetic Analyses

The sequences were edited and compared with other sequences deposited in the GenBank NCBI database using the BLAST tool [32]. An alignment of our sequences with those of other *Oscheius* species was performed for each amplified locus (ITS, SSU and LSU) using the default ClustalW parameters in MEGA 7.0 [33] and was optimized manually in BioEdit [34]. The final alignment lengths were 904 bp (ITS), 476 bp (D2D3) and 1565 bp (SSU). Pairwise distances were computed using MEGA 7.0 [33] in order to compare the ITS rDNA locus of the selected *Oscheius* species from the “*insectivorus*” group.

The phylogenetic reconstruction was performed from the datasets using the Bayesian inference (BI). All of the characters were treated as being equally weighted. *Heterorhabditis bacteriophora* Poinar, 1976; *H. zealandica* Poinar, 1990; and *H. downesi* Stock, Griffin & Burnell, 2002; were used as outgroup taxa. Bayesian analyses were performed in MrBayes 3.2.7 [35]. The best fit model was identified as the GTR + G model test using the MrModeltest 2.0 program [36]. Metropolis-coupled Markov chains Monte Carlo (MCMCMC) generations were run for 1 × 10^7^ cycles, and 1 tree was retained every 1000 generations. The Bayesian trees were visualized using the Mesquite program [37].

### 2.6. Analysis of the Cytochrome Oxidase I Gene

For the analysis, *Ocheius siddiqii* CS42 and five other *Oscheius* species or strains from our collection (Table 3) were used. The DNA was isolated from single females, and the cytochrome oxidase subunit I (COI) gene was amplified using the universal primers LCO-1490 (5′-GGTCAACAAATCATAAAGATATTGG-3′) (forward) and HCO-2198 (5′-TAAACTTCAGGGTGACCAAAAAATCA-3′) (reverse) [38] with the following PCR profile: 1 cycle of 94 °C for 2 min followed by 37 cycles of 94 °C for 30 s, 51 °C for 45 s, 72 °C for 2 min, and a final extension at 72 °C for 2 min. Sequences were deposited in GenBank under accession numbers OK142792-OK142797 (Table 3).

## 3. Results

**Figure 1:**
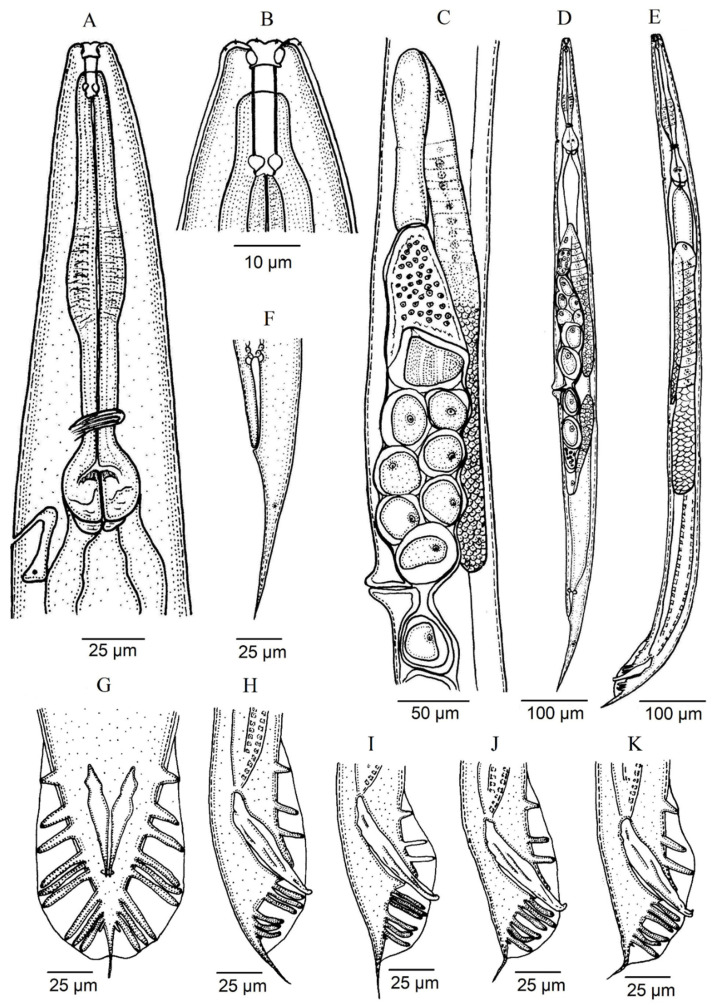
*Oscheius siddiqii* Tabassum and Shahina, 2010 (line drawing). (**A**): neck; (**B**): anterior end; (**C**): female reproductive system; (**D**): entire female; (**E**): entire male; (**F**): female posterior end; (**G**): male posterior end at ventral view; (**H**–**K**): male posterior end at lateral view showing variations in spicules.

**Figure 2:**
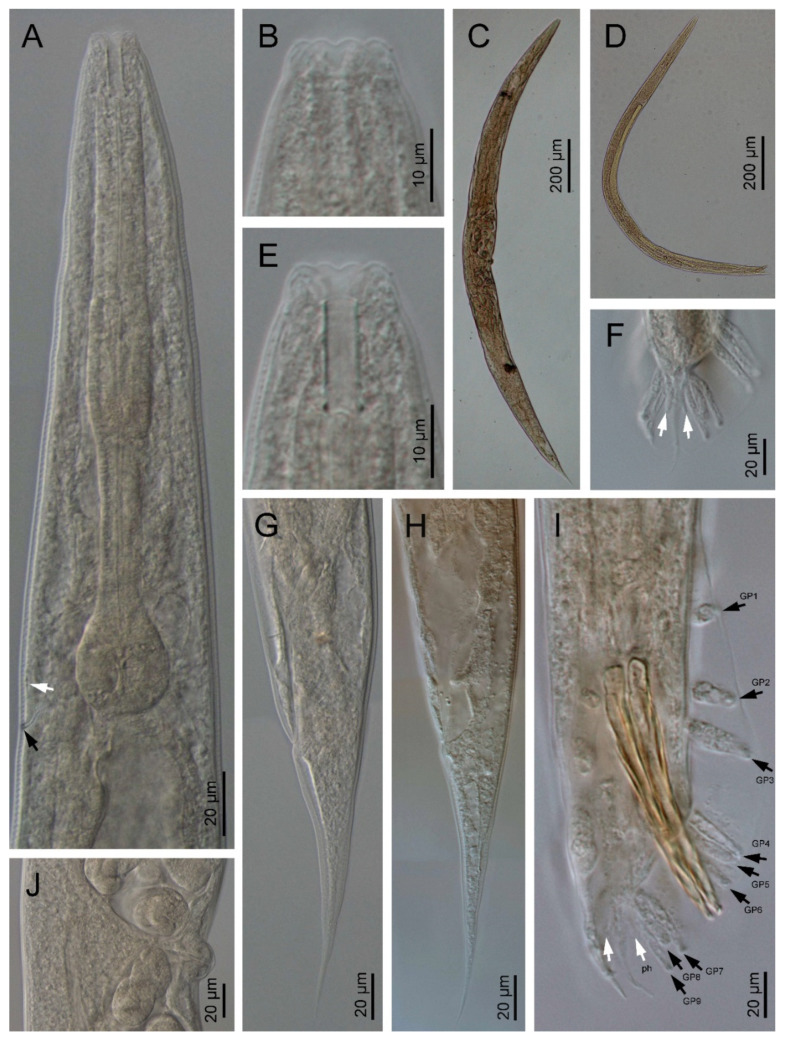
*Oscheius siddiqii* Tabassum and Shahina, 2010 (light microscopy). (**A**): Neck (black arrow pointing the excretory pore and white arrow pointing the hemizonid); (**B**,**E**): anterior end; (**C**): entire female; (**D**): entire male; (**F**): male tail end (arrows pointing phasmids); (**G**,**H**): female posterior end (arrow pointing the phasmid); (**I**): male posterior end (black arrows pointing genital papillae (GP), white arrows pointing phasmids (ph)); (**J**): female vulva.

**Figure 3:**
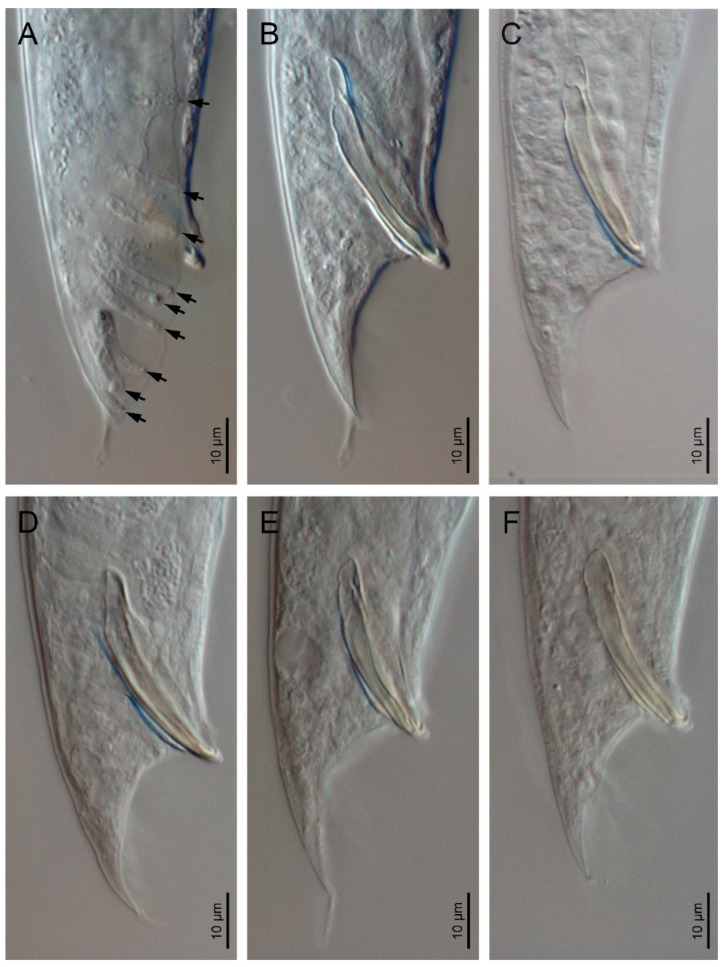
*Oscheius siddiqii* Tabassum and Shahina, 2010 (light microscopy). (**A**): Male posterior end at ventral view showing pattern of the genital papillae; (**B**–**F**): male posterior end at lateral view showing variations in spicules.

**Figure 4:**
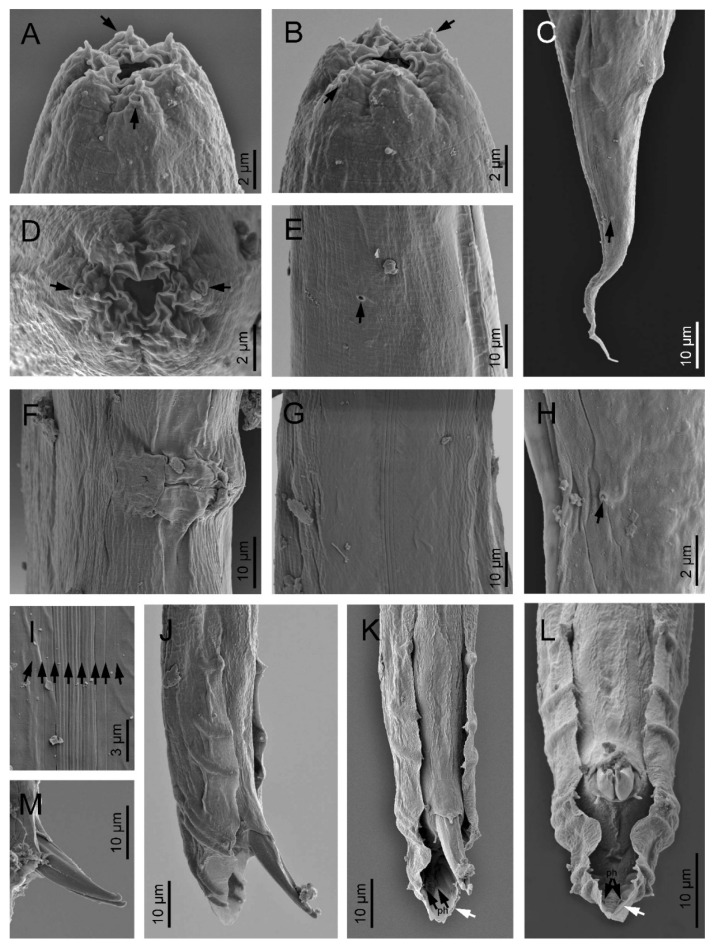
*Oscheius siddiqii* Tabassum and Shahina, 2010 (scanning electron microscopy). (**A**,**B**,**D**): Lip region in lateral (**A**,**B**) and frontal (**D**) views (arrows pointing the amphids); (**C**): female posterior end (arrow pointing the phasmid); (**E**): excretory pore (arrow); (**F**,**G**,**H**): vulva; (**I**): lateral field (arrows pointing the longitudinal incisures); (**J**,**K,L**): male posterior end in right lateral, subventral, and ventral views, respectively (black arrows pointing the phasmids (ph), white arrow pointing the filiform part of tail); (**M**): spicule tips.

*Oscheius siddiqii* Tabassum and Shahina, 2010 (Figure 1, Figure 2, Figure 3 and Figure 4, Table 1 and Table 2).

### 3.1. Description of Oscheius (Oscheius) siddiqii Tabassum & Shahina, 2010

Female (*n* = 20): Body larger than in males; 1.10–1.70 mm long; straight to slightly arcuate. Cuticle moderately transversely annulated. Lateral fields consisting of six distinct longitudinal incisures and other two sublateral poorly marked incisures. Lip region bearing six separate lips, with six inner long acute labial papillae and four outer short acute cephalic papillae in total; primary axils deeper than secondary axils. Amphidial apertures are pore-like and ovoid. Stoma rhabditoid type; 4.1–5.9 times longer than wide and 3.4–6.2 times the lip region width: conspicuous cheilostom, poorly cuticularised rounded rhabdia; gymnostom and pro-mesostegostom cuticularised with straight tubular rhabdia; short metastegostom with well-developed glottoid apparatus; short telostegostom with rounded cuticularised rhabdia. Conspicuous deirids present at 81–93% of neck length. Pharynx differentiated into cylindrical procorpus with swollen and subcylindrical metacorpus that is about twice as long as it is wide; long isthmus that is narrower than the procorpus; ovoid basal bulb with conspicuous valvular apparatus. Nerve ring encircling the isthmus at 70–89% of the neck length. Excretory pore at basal bulb or posterior that is located at 100–110% of the neck length. Conspicuous hemizonid just above excretory pore at the level of the basal bulb. Intestine with the cardiac region with thinner walls.

Didelphic and amphidelphic reproductive system; well-developed ovaries that are dorsally reflexed and that extend beyond the vulva; anterior and posterior ovaries on the same side of the intestine, with the anterior one larger than posterior one; oviducts with spermathecae are ovoid and filled with sperm and connected to dilated ovaries; long uteri that are divided in two parts are only observed in young females and comprise one distal tubular part and other proximal swollen part with thinner walls; uterine eggs in different embryonation stages compacted in uteri ranging in number from 6–24 and measuring 26–57 × 27–32 µm; vagina straight or slightly arcuate, ventral with lateral epiptygmata. Distinct rectum distinct that is 1.9–2.8 times anal body width, with three unicellular glands at its junction with the intestine. Large anus that is directed posteriorly. Straight, conoid tail with acute terminus. Distinct, pore like, phasmids located at 50–80% of tail length.

Male (*n* = 20): Body 0.92–1.44 mm long; “J”-shaped after heat killing with general morphology similar to female except for smaller size and arcuate posterior body. Monorchic reproductive system with testis ventrally reflexed anteriorly on the left side of intestine. Vas deferens are broad and light tubes filled with sperm and lack demarcation of seminal vesicle. Ejaculatory glands not present. Tail with conoid anterior part that is flanked by the bursa, and short and thin posterior filiform section that is located outside of the bursa and that is sometimes inconspicuous under LM. Bursa leptoderan with nine pairs of genital papillae (1 + 1 + 1/3 + 3 + ph): three pairs pre-cloacal (GP1–GP3) and six pairs post-cloacal, with three pairs at mid tail length (GP4–GP6) and three pairs (GP7–GP9) at the terminal length; GP1, GP2, and GP3 are spaced apart; space between GP1 and GP2 is greater than the space between GP2 and GP3. Phasmids are easily observed and are located posterior to GP9 at 55–65% of the tail length. Long spicules that are broad and arcuate as well as larger than the gubernaculum and a manubrium that is ventrally bent and conoid to rounded; short calamus and lamina that are slightly ventrally curved with dorsal hump that is variable in size; long ventral velum and hooked tip. Gubernaculum with manubrium-corpus that is almost straight; well-developed crura with acute tip that is at 37–49% of the spicule length.

Third stage juvenile (J3) (*n* = 20): Robust body that is 0.50–0.60 mm long with straight or slightly curved at posterior end. Cuticle is almost smooth, with a lip region similar to adult specimens. Narrow stoma that 2.1–3.0 times as narrow as the lip region is wide. Pharynx clearly visible and differentiated into the three rhabditoid parts. Nerve ring surrounding the isthmus that is about 60–74% of the neck length. Excretory pore at or just posterior to basal bulb. Obscure deirid. Reduced cardia reduced that is surrounded by intestinal tissue. Rectum longer than anal body width. Anus prominent. Tail conoid; spicate with fine hair-like terminus without mucro with terminal hyaline part that is 42–66% of the tail length.

### 3.2. Molecular Characterization

*Oscheius siddiqii* is characterized by sequences of the ITS region, the SSU and D2D3 region of LSU of the rDNA and mitochondrial cytochrome oxidase I gene. Two variants of the ITS sequence were observed in the strain CS42, which had either C (40%) or T (60%) base at a position, which corresponded to position 335 bp of the sequence MT835467.1.

The SSU sequence of *O. siddiqii* was the most similar to those of *O. myriophilus* Poinar, 1986 (approximately 89–90%) and *O. safricanus* Serepa-Dlamini & Gray 2018 (name amended from *O. safricana*) (98.7%) (Table 4). In the ITS region, the highest similarities between the *O. siddiqii* sequences were observed with *O. citri* Tabassum, Shahina, Nasira and Erum, 2016 (81.5%); *O. myriophilus* (80.4%); and *O. chongmingensis* Zhang, Liu, Xu, Sun, Yang, An, Gao, Lin, Lai, He, Wu & Zhang, 2008 (79.9%) (Table 5).

The analysis of the cytochrome oxidase I gene showed that the sequence of *O. siddiqii* differs from other available species from the “*insectivorus*” group by 3–9%, with *O. chongmingensis* being the most similar (97.3%).

**Figure 5:**
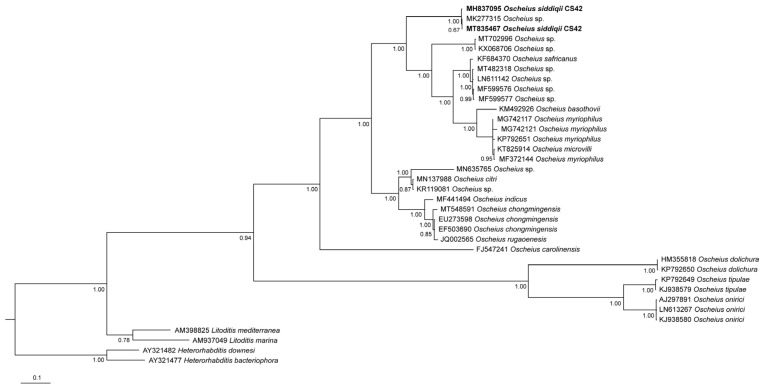
Phylogenetic relationships of *Oscheius siddiqii* and other *Oscheius* species as inferred from Bayesian analysis of sequences of the internal transcribed spacer (ITS1-5.8S-ITS2) rDNA segment. Bayesian posterior probabilities (%) higher or equal to 60% are provided at each node. The scale bar indicates the number of substitutions per site.

**Figure 6:**
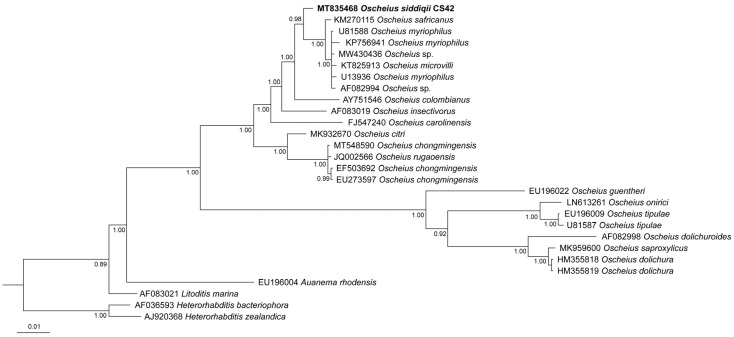
Phylogenetic relationships of *Oscheius siddiqii* and other *Oscheius* species as inferred from Bayesian analysis of sequences of the small subunit (18S) of the rDNA segment. Bayesian posterior probabilities (%) that are higher than or equal to 60% are provided at each node. The scale bar indicates the number of substitutions per site.

**Figure 7:**
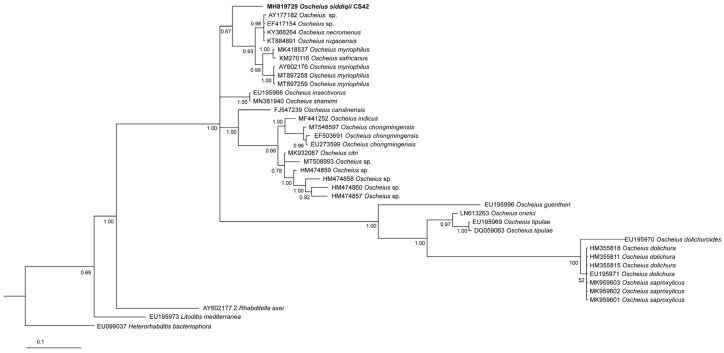
Phylogenetic relationships of *Oscheius siddiqii* and other *Oscheius* species as inferred from Bayesian analysis of sequences of the D2–D3 fragments of large subunit (28S) of rDNA region. Bayesian posterior probabilities (%) higher than or equal to 60% are provided at each node. The scale bar indicates the number of substitutions per site.

All of the phylogenetic analyses show that *O. siddiqii* is a sister taxon of the group consisting of *O. myriophilus*; *O. safricanus*; *O. microvilli* Zhou, Yang, Wang, Bao, Wang, Hou, Lin, Yedid & Zhang, 2017; and several unidentified *Oscheius* species (Figure 5, Figure 6 and Figure 7). Interestingly, the ITS tree shows *O. siddiqii* CS42 forming a highly supported monophyletic clade with *Oscheius* sp. (MK277315), which was also collected from India, their sequences being almost identical (similarity over 99.1%); these nematodes are most likely conspecific.

## 4. Discussion

### 4.1. Remarks

Unfortunately, some of the currently recognized *Oscheius* species have inadequate or no molecular support. This is because at present, since predominantly molecular identification of nematodes is used, the lack of molecular data for nematode species complicates the systematics of the group.

Generally, the species with no molecular data not only complicate systematic and phylogenetic studies but also present a problem for biodiversity studies, and it is thus desirable to match these older taxa with molecular data. This, however, presents a challenge, especially in the case of nematodes, where a number of cryptic species have been reported [39,40,41], and therefore, assigning the molecular data just based on morphological similarities poses an inherent risk of overlooking cryptic species. Nevertheless, such a situation can be possibly mended in the future if the original species is re-isolated. Alternatively, there will be more taxa than the actual evolutionary lineages, and according to Carstens et al. [42], in most contexts, it is better to fail to delimit species than it is to falsely delimit entities that do not represent actual evolutionary lineages. Moreover, *Oscheius* species, for which both morphology and molecular data are available, show interspecific differences in both datasets, and so far, no cryptic species have been reported in this group.

The material examined now from India agrees well with *O. siddiqii* and *O. niazii* Tabassum & Shahina, 2010 (the correct name of the latter should be *O. niazensis* because it refers to a village), the two very similar species that were both described from the same district in Pakistan. Regrettably, we were not able to gain the access to paratypes of these Pakistani taxa in order to study their morphology and to corroborate their identity despite numerous attempts. The descriptions of the type populations of both Pakistani species, despite the poor quality of their illustrations and the existence of some inaccuracies in the drawings, show negligible morphological and morphometrical differences (see Supplementary Tables S2 and S3), and the measurements mainly overlap, with *O. niazensis* having somewhat larger ranges.

Therefore, with no significant morphological difference between both nematodes, we consider them to be two populations of a single species. Alternatively, the Pakistani and Indian populations could represent two morphologically identical species; however, we consider this possibility to be unlikely. Comparisons of the Indian populations examined now with both Pakistani species have shown that they resemble each other in terms of all of the important traits, such as stoma morphology (especially in *O. niazensis*), the arrangement of the genital papillae, with the GP2 and GP3 being spaced closer together, and the spicules, with the ventral bent tip, with no other important differences being detected. Unfortunately, the Pakistani species lack molecular data in order to adequately compare them to the Indian populations. Nevertheless, the morphological and morphometrical data show that the Indian populations and both Pakistani populations are the same species. According to the International Code of Zoological Nomenclature (ICZN), *O. siddiqii,* being the first described species in the paper published by Tabassum & Shahina [16], is the valid name for the taxon and the type population, whereas *O. niazensis* should be considered as its junior synonym.

For the first time, we provide the COI sequences for five *Oscheius* species, and these data will form the basis for further molecular systematic studies of the group. The sequence diversity among species was lower than it was in the ITS sequences, which is in agreement with the observations made with, e.g., heterorhabditid entomopathogenic nematodes [43] or populations of the parasitic nematode *Camallanus cotti* [44]. Nevertheless, the COI sequences show both interspecific and intraspecific variation and thus provide further resolution among *Oscheius* species.

### 4.2. Diagnosis (Based on Indian Populations)

*Oscheius siddiqii* is characterized by its body size, which ranges from 1.10–1.70 mm in females and from 0.92–1.44 mm in males; a cuticle with delicate transverse striations; lateral fields consisting of eight prominent lines; a lip region bearing six separate lips; a tubular stoma that is 13–20 µm in length; a pharynx with a more robust and subcylindrical metacorpus; a nerve ring at the isthmus–bulb junction; an excretory pore situated at the level of the basal pharyngeal bulb or slightly posterior; a didelphic–amphidelphic female reproductive system ; an equatorial vulva (V  =  45–57) with a lateral epiptygmata; a rectum that is 1.9–2.8 times the anal body width; a female conical tail with an acute tip (123–180 µm long, *c* = 7.8–15.4, *c’* = 3.5–7.5) and a male conoid tail (36–49 µm long, *c* = 20.0–33.0, *c’* = 1.4–2.5) with small filiform part, spicules 39–55 µm long with a crochet-like tip; and a gubernaculum that is 21–26 µm long. A bursa leptoderan with nine pairs of genital papillae (GP) (1 + 1 + 1/3 + 3 + ph) and well developed phasmids are located posteriorly to GP9.

### 4.3. Phylogenetic Relationships of the Species of the Subgenus Oscheius

The reliable reconstruction of phylogenetic relationships within the subgenus *Oscheius* is hampered by the fact that for some established species, not all standardly used genetic markers are available. This not only complicates phylogenetic reconstructions of the relationship within the group but also presents a challenge for distinguishing the newly described species from the existing ones. In our analyses, the position of the majority of *Oscheius* species was consistent in all trees, and the majority of species were clearly separated from other species, with four prominent exceptions: *Oscheius basothovii* Lephoto & Gray, 2019; *O. safricanus*, *O. rugaoensis* Zhang, Liu, Tan, Wang, Qiao, Yedid, Dai, Qiu, Yan, Tan, Su, Lai & Gao, 2012; and *O. microvilli* (Figure 5, Figure 6 and Figure 7). Below, we revise the available molecular and morphological data of these taxa. In the D2D3 tree, the sequence attributed to the *O. shamimi* Tahseen & Nisa, 2006 (MN381940) groups with *O. insectivorus* (Körner, 1954) Andrássy, 1976 (EU195968) with 100% support, and both sequences are almost identical, with only one bp deletion difference. The D2D3 region is known to provide little resolution, and identical D2D3 sequences were observed in some closely related *Meloidogyne* species [45]. However, considering the fact that intraspecific differences in *Oscheius* species with more available D2D3 sequences (*O. Myriophilus*, *O. chongmingensis*) are significantly higher and range up to 13 bp, we assume that most likely, the sequence MN381940 actually belongs to *O. insectivorus*.

#### 4.3.1. *Oscheius basothovii* and *O. safricanus*

Based on the analyses of the ITS and SSU sequences, *Oscheius safricanus* seems to be very closely related to *O. myriophilus,* and in the LSU tree, it falls within *O. myriophilus* strains. Unfortunately, it is not possible to compare the morphology because the line illustration provided by Serepa-Dlamini & Gray [46] is slightly modified from that of the description of *Oscheius rugaoensis* provided by Zhang et al. [47], and surprisingly, the LM micrographs, at least in the male (see Figure 3B), apparently correspond with some species of the genus *Steinernema.* According to the morphology of the spicules.

Interestingly, the ITS tree shows that *Oscheius basothovii* is even closer to *O. myriophilus,* with which it forms a monophyletic clade that is sister to *O. safricanus*. Unfortunately, no LSU and SSU sequences have been given in the description of *O. basothovii* [48], so it is not possible to assess the status of this species. Morphologically, the very poor quality of the illustrations provided for *O. basothovii* by Lephoto & Gray [48] makes it impossible to correctly evaluate its morphology and to compare it with *O. myriophilus*.

Both species, *O. basothovii* and *O. safricanus*, could be considered as *species inquirendae*.

#### 4.3.2. *Oscheius microvilli*

The original description of *O. microvilli* only provides the ITS and SSU sequences under accession numbers KT825914 and KT825913, respectively [49]. In both of our phylogenetic reconstructions, *O microvilli* falls into the clade of other *O. myriophilus* strains with high support.

Furthermore, the differences between the ITS sequence attributed to *O. microvilli* and the respective sequences of other *O. myriophilus* are negligible and are within the standard intraspecific range (Table 5). In the case of SSU, the analysis revealed more than 60 differences (Table 4). The fact that the conservative SSU harbors several times more differences than the variable ITS suggests that the SSU sequence might be inadequately edited. A closer look at the alignment shows that all of the differences occur at the beginning (1–41 bp) and at the end (1572–1615 bp) of the sequence and in positions 1209–1332 bp. In all of the other positions, the sequence is identical to the sequences of the *O. myriophilus* strains. The corresponding positions of the alignment are quite conservative, and these variable positions in the sequence KT825913 are probably sequencing errors. To conclude, the available molecular data attributed to *O. microvilli* are not sufficient to separate this taxon from *O. myriophilus*. Morphological comparisons of these two species do not display significant differences, with the exception of males, but the male morphology described in the description of *O. microvilli* agrees with *Caenorhabditis sinica* according the bursa morphology among other morphological characteristics and morphometry (see [4]). Therefore, *O. microvilli* is proposed as a junior synonym of *O. myriophilus*.

#### 4.3.3. *Oscheius rugaoensis*

The original description of *O. rugaoensis* provided the ITS and SSU sequences [47], and in both the ITS and SSU trees, this species is a member of a monophyletic clade together with several strains of *O. chongmingensis*, suggesting that these nematodes are in fact conspecific. Moreover, distance analyses of the ITS and SSU sequences of *O. rugaoensis* and *O. chongmingensis* only show an insignificant number of differences (Table 4 and Table 5). The NCBI GenBank database contains one D2D3 sequence attributed to *O. rugaoensis* (KT884891) that is, however, almost identical to the sequence attributed to *O. necromenus* (Sudhaus & Schulte, 1989), and in the LSU tree, their position strongly differs from the position of *O. rugaoensis* in both ITS and SSU trees. This sequence thus probably belongs to *O. necromenus*. In general, the available molecular data fail to support *O. rugaoensis* as a separate species from *O. chongmingensis*. Morphologically, *O. rugaoensis* shows a resemblance to *O. chongmingensis,* mainly in the morphology of the stoma, the position of the excretory pore and nerve ring, the morphology of both the female and male tails and spicules, the presence of pseudopeloderan type bursa, and the presence of small bristle-like sensilla posterior to the cloacal opening. Morphometrically, the also show close similitude to each other, such as in the size of the females; similar ratios (a, b, c, and c’); similar anal body width; similar SW%, GS% and V%; and other morphometric measurements (Supplementary Tables S2 and S3). Therefore, *O. rugaoensis* is proposed as a junior synonym of *O. chongmingensis*, while the population from Japan [50] attributed to *O. rugaoensis* should be considered conspecific with the population of *O. necromenus* that is described from Iran [51].

## 5. Conclusions

Potentially entomopathogenic nematodes of the genus *Oscheius* have been the subject of increased scientific interest in recent years. However, the research is complicated by unclear taxonomy, with some of the species being poorly described and having inadequate or no molecular support. In our study, we provide a morphological and molecular characterization of *Oscheius siddiqii* and propose *O. niazensis* as its junior synonym.

Using the available molecular data and newly sequenced COI gene of five *Oscheius*, we revised other species belonging to the genus *Oscheius* and discuss species delimitation. Our results show that a majority of *Oscheius* with the available molecular support are well characterized by the combination of traditional rDNA genes (ITS, LSU and SSU) but that the COI sequence provides further resolution among *Oscheius* species. Based on morphological and molecular data, we propose *O. microvilii* and *O. rugaoensis* as junior synonyms of *O. myriophilus* and *O. chongmingensis*, respectively. Furthermore, *O. basothovii* and *O. safricanus* should be considered as *species inquirendae.* Our results highlight the importance of the proper molecular characterisation of newly described *Oscheius* species. Therefore, any future species description should provide all three standardly used rDNA markers (ITS, SSU, and LSU) in combination with at least one mitochondrial COI gene.

## Figures and Tables

**Table 1:** Morphometric data of *Oscheius siddiqii* Tabassum and Shahina, 2010 (isolate CS42). All measurements are in µm (except *n*, ratio and percentage) and in the form of mean ± SD (range).

Characters	Female	Male	J3 Juvenile
*n*	20	20	20
Body length (L)	1465 ± 135 (1204–1697)	1135 ± 126 (916–1441)	534 ± 21 (502–574)
*a* (L/MBD)	16.7 ± 1.2 (14.2–19.4)	24 ± 4.7 (14.7–25.8)	21 ± 1.0 (19.3–22.7)
*b* (L/NL)	7.7 ± 0.9 (6.1–8.9)	6.7 ± 0.6 (5.3–7.7)	4.3 ± 0.3 (3.7–4.9)
*c* (L/T)	10.4 ± 1.5 (7.8–15.4)	28 ± 3.1 (20.1–32.9)	7.4 ± 0.6 (7.0–8.2)
*c’* (T/ABW)	4.8 ± 0.8 (3.5–6.3)	1.8 ± 0.2 (1.5–2.5)	6.0 ± 0.7 (4.9–7.2)
*V*% (AV/L × 100)	50 ± 3.1 (45–57)	–	–
Lip region width	7.1 ± 0.6 (6–8)	7.1 ± 0.8 (6–9)	4.1 ± 0.8 (3–6)
Stoma length	17.1 ± 1.1 (15–20)	17.1 ± 2.1 (13–20)	13.9 ± 1.6 (10–17)
Stomatal tube width	3.1 ± 0.3 (2.5–3.8)	4.0 ± 0.7 (3.2–5.4)	–
Procorpus length	64 ± 3.4 (58–69)	58 ± 5.6 (47–66)	42 ± 2.6 (37–46)
Metacorpus length	38 ± 2.9 (32–42)	33 ± 1.6 (31–36)	21 ± 2.1 (18–23)
Isthmus length	64 ± 3.4 (58–69)	45 ± 3.3.6 (37–50)	29 ± 2.2 (37–46)
Bulb length	35 ± 3.5 (24–34)	31 ± 3.0 (27–37)	21 ± 2.1 (18–23)
Pharynx length	183 ± 6.6 (169–198)	165 ± 7.3 (139–173)	106 ± 7.5 (95–127)
Nerve ring—ant. end (NR)	153 ± 10.6 (133–175)	138 ± 10.6 (119–178)	73 ± 6.9 (61–81)
Excretory pore– ant. end (EP)	191 ± 17 (171–224)	189 ± 22.4 (162–212)	120 ± 6.6 (100–132)
Deirid–ant. end	163 ± 13.0 (141–196)	135 ± 13.6 (106–156)	–
Neck length (stoma + pharynx, NL)	221 ± 8.0 (205–239)	183± 7.2 (172–192)	125 ± 4.8 (116–136)
Body width at neck base	54 ± 6.3 (42–66)	42 ± 3.8 (36–51)	28 ± 2.1 (25–31)
Mid-body diameter (MBD)	94 ±7.5 (81–114)	49 ± 7.7 (38–64)	25 ± 1.4 (23–28)
Uterus or testis length	69 ± 15.5 (53–87)	557 ± 36 (464–596)	–
Anterior spermatheca length	44 ± 8.0 (31–63)	–	–
Anterior genital branch	313 ± 50 (213–392)	–	–
Posterior spermatheca length	39 ± 7.4 (31–54)	–	–
Posterior genital branch	257 ± 40 (202–329)	–	–
Vagina length	24 ± 4.1 (18–31)	–	–
Vulva—ant. end (AV)	732 ± 57 (610–860)	–	–
Rectum length	70 ± 9.8 (57–81)	–	31 ± 2.6 (26–39)
Anal body diam. (ABD)	32 ± 2.9 (26–41)	21 ± 2.9 (19–26)	12.8 ± 1.3 (11–16)
Tail length (T)	147 ± 20 (123–169)	41 ± 3.3 (38–48)	76 ± 10.1 (59–98)
Phasmid to anus distance	42 ± 6.3 (33–57)	24 ± 1.2 (21–26)	–
Spicule length (SL)	–	44 ± 5.1 (39–53)	–
Gubernaculum length (GL)	–	21 ± 3.1 (22–26)	–
Hyaline part of tail (H)	–	–	41 ± 6.3 (36–49)

– = characters absent or not measured.

**Table 2:** Morphometrics of *Oscheius siddiqii* Tabassum and Shahina, 2010 (isolation CS43). All measurements are in µm (except *n*, ratio and percentage) and in the form of mean ± SD (range).

Characters	Female	Male	J3 Juvenile
*n*	20	20	20
Body length (L)	1439 ± 139 (1121–1586)	1021 ± 77 (920–1180)	551 ± 28 (500–598)
*a* (L/MBD)	16.9 ± 1.8 (13.6–19.2)	23 ± 2.1 (19.9–26.7)	19.9 ± 1.3 (17.7–22.4)
*b* (L/NL)	72 ± 0.9 (5.6–8.2)	6.0 ± 0.6 (5.1–7.2)	4.2 ± 0.2 (3.8–4.8)
*c* (L/T)	8.3 ± 0.8 (6.3–9.5)	25 ± 2.5 (19.7–30.5)	6.9 ± 0.7 (5.7–8.3)
*c’* (T/ABD)	6.6 ± 0.7 (5.2–7.5)	1.8 ± 0.3 (1.4–2.5)	5.9 ± 0.7 (4.8–7.7)
*V*% (AV/L × 100)	52 ± 11.2 (48–61)	–	–
Lip region width	6.1 ± 0.6 (6–7)	5.8 ± 0.8 (5–7)	3.5 ± 0.6 (2–5)
Stoma length	15.9 ± 1.6 (13–19)	14.9 ± 1.6 (13–20)	13.8 ± 2.0 (10–17)
Stomatal tube width	3.2 ± 0.8 (2–5)	3.1 ± 0.5 (2–4)	–
Procorpus length	61 ± 6.0 (53–68)	54 ± 6.1 (45–69)	41 ± 1.4 (37–48)
Metacorpus length	36 ± 3.3 (31–41)	32 ± 3.4 (27–39)	24 ± 1.6 (21–27)
Isthmus length	50 ± 2.3 (46–53)	38 ± 5.0 (44–48)	33 ± 3.1 (30–41)
Bulb length	30 ± 2.0 (28–34)	29 ± 3.0 (24–35)	18.9 ± 1.7 (17–24)
Pharynx length	177 ± 4.7 (169–183)	153 ± 9.8 (139–173)	117 ± 4.9 (108–129)
Nerve ring—ant. end (NR)	154 ± 12.9 (138–181)	149 ± 8.0 (130–162)	82 ± 4.9 (70–89)
Excretory pore—ant. end (EP)	197 ± 13 (172–216)	195 ± 14.1 (162–220)	118 ± 5.6 (108–129)
Deirid—ant. end	159 ±10.0 (139–170)	133 ± 8.4 (118–149)	–
Neck length (stoma + pharynx, NL)	196 ± 4.4 (189–201)	171 ± 10.1 (158–190)	131 ± 4.7 (123–143)
Body width at neck base	34 ± 2.7 (30–38)	35 ± 4.8 (26–42)	27 ± 2.6 (23–34)
Mid-body diameter (MBD)	81 ± 12 (72–98)	44 ± 5.6 (38–58)	28 ± 1.4 (25–30)
Uterus or testis length	74.1 ± 15.3 (56–98)	539 ± 40 (545–590)	–
Anterior spermatheca length	38 ± 5.1 (32–49)	–	–
Anterior genital branch	275 ± 41 (210–343)	–	–
Posterior spermatheca length	37 ± 5.0 (28–44)	–	–
Posterior genital branch	240 ± 30 (204–290)	–	–
Vagina length	23 ± 2.6 (19–28)	–	–
Body width at vulva	77 ± 11.2 (59–109)	–	–
Vulva—ant. end (AV)	741 ± 74 (568–829)	–	–
Rectum length	67 ± 4.6 (61–75)	–	35 ± 2.8 (28–39)
Anal body diam. (ABD)	27 ± 3.0 (22–34)	23 ± 1.9 (20–27)	14 ± 1.5 (11–16)
Tail length (T)	173 ± 6.2 (154–180)	42 ± 4.7 (36–49)	81 ± 7.7 (70–96)
Phasmid to anus distance	39 ± 4.8 (34–50)	22 ± 3.6 (17–26)	–
Spicule length (SL)	–	51 ± 4.9 (37–55)	–
Gubernaculum length (GL)	–	25 ± 2.7 (21–26)	–
Hyaline part of tail (H)	–	–	42 ± 3.2 (36–47)

– = characters absent or not measured.

**Table 3:** Pairwise distances of the COI gene among selected *Oscheius* species. The values above diagonal represent percent similarities, and the values below diagonal represent numbers of character differences.

S. No.	COI Region	1	2	3	4	5	6	7	8	9	10	11
1	OK142792 *O. siddiqii* CS42		92.9	92.9	91.0	97.3	89.1	89.4	88.5	89.9	88.1	88.1
2	OK142795 *O. myriophilus* 1b	44		99.9	89.7	92.5	89.2	89.8	89.7	90.2	88.7	88.7
3	OK142794 *O. myriophilus* JU1386	44	1		89.6	92.6	89.4	89.4	89.2	89.7	88.3	88.3
4	OK142796 *O. citri* WGN	56	64	65		90.4	86.8	89.4	88.1	88.9	87.3	87.3
5	OK142793 *O. chongmingensis*	17	51	57	60		88.9	89.2	88.4	89.5	88.1	88.1
6	OK142797 *O. guentheri*	68	73	81	82	85		88.0	87.4	88.3	87.4	87.4
7	MF196100.1 *O. onirici* 16-33834	46	50	55	46	56	62		99.2	99.7	98.6	98.6
8	MK754223.1 *O. onirici* N6691	48	49	54	50	58	63	5		99.7	98.4	98.4
9	KY582595.1 *O. onirici* Wisconsin 6	40	44	49	44	50	56	2	2		98.3	98.3
10	LN613269.1 *O. onirici* FDL-2014	44	48	53	47	54	57	8	10	10		100
11	LN613268.1 *O. onirici* FDL-2014	44	48	53	47	54	57	8	10	10	0	

**Table 4:** Pairwise distances of the *SSU* region of the *rDNA* among species of the “*insectivorus*” group of the genus *Oscheius*. The values above the diagonal represent percent similarities, and values below the diagonal represent numbers of character differences.

S. No.	SSU Region	1	2	3	4	5	6	7	8	9	10	11	12	13	14	15	16
1	U81588 *O. myriophilus*		99.8	100	99.8	96.0	99.9	99.6	96.9	97.1	96.8	96.7	98.9	97.5	97.7	97.0	96.4
2	KP756941 *O. myriophilus*	3		99.7	99.6	95.8	99.8	99.3	96.7	96.9	96.7	96.5	98.7	97.3	97.5	96.8	96.2
3	MW430436.1 *O. myriophilus*	0	3		100	98.2	99.9	99.7	96.8	96.8	96.8	96.7	99.0	97.2	98.5	97.1	97.9
4	U13936 *O. myriophilus*	3	6	0		95.9	99.8	99.6	96.8	96.9	96.7	96.6	98.8	97.4	97.6	96.9	96.3
5	KT825913 *O. microvilli*	63	66	17	65		95.9	97.8	93.7	94.7	93.7	93.7	96.0	93.9	93.9	94.7	93.1
6	AF082994 *O. myriophilus*	1	4	1	4	64		99.6	96.8	97.0	96.8	96.7	98.9	97.5	97.7	97.0	96.4
7	KM270115 *O. safricanus*	4	7	2	4	22	4		96.3	96.3	96.2	96.2	98.7	96.9	97.0	96.6	96.9
8	MT548590 *O. chongmingensis*	51	54	30	53	98	52	37		99.9	99.9	99.9	96.4	96.5	96.2	98.3	95.9
9	EF503692 *O. chongmingensis*	45	48	30	47	82	46	37	1		100	99.9	96.7	96.5	96.3	98.4	95.9
10	EU273597 *O. chongmingensis*	53	56	30	55	99	54	38	1	0		99.9	96.3	96.3	95.9	98.3	95.8
11	JQ002566 *O. rugaoensis*	52	55	31	54	99	53	38	1	2	2		96.3	96.3	96.0	98.3	95.7
12	MT835468 *O. siddiqii* CS42	16	19	9	18	60	17	12	55	49	56	56		98.0	98.3	97.1	97.0
13	AF083019 *O. insectivorus*	42	45	26	43	96	43	31	58	54	64	59	30		97.0	96.8	96.4
14	AY751546 *O. colombianus*	38	41	14	40	95	39	30	63	57	69	64	26	50		96.6	96.8
15	MK932670 *O. citri* WGN	46	49	27	48	81	47	33	26	24	27	27	44	50	52		96.1
16	FJ547240 *O. carolinensis*	60	63	20	62	108	61	31	68	63	71	69	46	60	53	60	

**Table 5:** Pairwise distances of the ITS region of the rDNA among species of the “*insectivorus*” group of the genus *Oscheius*. Values above the diagonal represent percent similarities, and values below the diagonal present numbers of character differences.

S. No.	ITS Region	1	2	3	4	5	6	7	8	9	10	11	12	13	14	15	16	17	18	19
1	MG742117 *O. myriophilus*		97.6	99.4	99.3	99.4	85.7	89.2	73.2	74.6	75.4	73.8	64.9	75.6	76.7	79.7	78.8	73.1	86.5	70.0
2	MG742121 *O. myriophilus*	23		97.6	97.6	98.2	84.4	88.6	73.9	74.1	74.9	74.1	64.1	75.7	76.1	79.9	79.7	72.3	85.3	69.4
3	KT825914 *O. microvilli*	6	23		99.3	99.5	85.7	89.3	74.2	74.8	75.6	74.1	64.9	76.1	76.7	80.1	79.5	72.8	86.5	69.8
4	KP792651 *O. myriophilus*	7	24	7		98.9	85.4	88.8	74.1	74.8	75.6	74.5	64.6	76.4	76.5	80.4	79.6	72.8	86.2	69.9
5	MF372144 *O. myriophilus*	5	15	4	9		85.8	90.6	72.7	72.8	72.9	72.6	65.6	72.8	76.9	77.6	77.7	72.6	86.3	68.7
6	KF684370 *O. safricanus*	115	125	115	117	112		83.8	72.4	72.6	72.7	72.4	67.3	73.3	76.9	77.7	77.7	74.1	98.5	67.6
7	KM492926 *O. basothovii*	86	91	85	89	73	122		72.2	72.3	72.4	72.2	66.7	72.9	75.5	74.7	74.8	72.5	84.1	69.7
8	MT548591 *O. chongmingensis*	260	247	245	250	216	217	214		98.2	98.6	98.1	76.0	88.8	77.4	79.5	79.9	93.3	74.1	69.1
9	EU273598 *O. chongmingensis*	238	244	237	237	215	215	213	18		99.9	98.7	76.3	89.4	77.1	79.0	79.0	93.7	74.3	68.5
10	EF503690 *O. chongmingensis*	228	234	227	227	214	214	212	14	1		99.1	76.4	89.5	77.2	79.2	79.2	93.8	74.4	68.9
11	JQ002565 *O. rugaoensis*	248	248	246	247	217	217	214	20	13	9		76.2	89.2	76.9	79.3	79.5	93.6	74.0	68.8
12	MN635765 *Oscheius* sp.	285	291	285	287	273	262	251	205	202	201	202		84.6	70.6	69.4	69.5	78.3	67.5	62.3
13	MN137988.2 *O. citri*	229	236	226	228	220	214	213	111	105	104	108	135		79.1	81.4	81.5	89.9	74.9	70.0
14	MK277315 *Oscheius* sp.	175	179	175	176	170	165	181	176	178	177	180	222	164		99.6	99.7	77.9	77.9	69.3
15	MT835467 *O. siddiqii* CS42	187	191	184	185	173	171	190	196	200	196	200	248	180	3		99.9	77.5	78.8	72.9
16	MH837095.2 *O. siddiqii* CS42	199	193	191	197	172	171	189	196	200	196	200	247	179	2	1		77.6	78.8	73.4
17	MF441494 *O. indicus*	217	223	219	219	214	200	207	57	53	52	54	178	86	171	185	184		75.4	66.4
18	LN611142 *Oscheius* sp.	113	123	113	115	111	12	125	212	210	209	212	262	209	165	169	169	198		69.1
19	FJ547241 *O. carolinensis*	290	293	290	291	256	263	237	309	309	302	309	325	294	238	254	253	282	259	

## Data Availability

Not applicable.

## Supplementary Materials

The following are available online at https://www.mdpi.com/article/10.3390/biology10121239/s1, Table S1: Diagnosis of the genus and list of species, Table S2: Comparative morphometrics of the females of the species of the subgenus *Oscheius*. All measurements in µm except for the indexes, Table S3: Comparative morphometrics of the males of the species of the subgenus *Oscheius*. All measurements in µm except for the indexes.
