# Supplementary material for: Morphological, Morphometrical and Molecular Characterization of Oscheius siddiqii Tabassum and Shahina, 2010 (Rhabditida, Rhabditidae) from India with Its Taxonomic Consequences for the Subgenus Oscheius Andrássy, 1976
Source: Biology (Basel). 2021 Nov 27;10(12):1239. doi: 10.3390/biology10121239 (PMC8698764; doi:10.3390/biology10121239)
Supplement: Supplementary file 1 [file biology-10-01239-s001.zip › Supplementary table S1.pdf]

Diagnosis of the genus and list of species (updated after Abolafia and Peña-Santiago, 2019)

***Oscheius* Andrássy, 1976**

= *Rhabditis* (*Oscheius* Andrássy, 1976) Sudhaus, 1993

= *Dolichorhabditis* Andrássy, 1983

= *Heterorhabditidoides* Zhang, Liu, Xu, Sun, Yang, An, Gao, Lin, Lai, He, Wu and Zhang, 2008

Diagnosis

Rhabditidae. Small- to medium-sized nematodes, 0.50-3.25 mm long. Lateral field with three to five ridges (four to six incisures). Lip region continuous. Stoma tubular, bearing glottoid apparatus with small elongate teeth. Pharynx consisting of cylindrical corpus gradually enlarging posteriorly, with not swollen metacorpus, and basal bulb bearing duplex haustrulum. Secretory-excretory duct elongated, looped and strongly sclerotized. Female genital system didelphic-amphidelphic, with equatorial vulva. Female rectum conspicuously longer than anal body diameter, proximally dilated, forming a bladder-like expansion of the hind gut (often filled with faeces). Female tail conical to conical elongate. Testis reflexed ventrally. Bursa peloderan or leptoderan, anteriorly open, with wide velum bearing nine genital papillae arranged 1+1+1/3+3, GP5 and GP8 opening dorsally (bursa formula: v1,v2,v3/v4,ad,v5–v6,pd,v7,ph). Male tail conoid with or without a short acute terminal tip out of the bursa. Phasmid posterior to the last GP, tubular. Spicules free, dagger-shaped, head and slanted shoulder, the tip thickened.

Subgenus *Oscheius* Andrássy, 1976

Diagnosis

Stoma tubular with metastegostom bearing warts. Bursa leptoderan. Male tail conoid with tip out of the bursa, filiform, variable in length. Spicules distally hook-shaped, like a crochet needle.

Type species

Oscheius (Oscheius) insectivorus (Körner, 1954) Andrássy, 1976

= *Rhabditis (Choriorhabditis) insectivora* Körner, 1954

= *Heterorhabditidoides (Oscheius) insectivora* (Körner, 1954) Zhang, Liu, Tan, Wang, Qiao, Yedid, Dai, Qiu, Yan, Tan, Su, Lai and Gao, 2012

Other species

Oscheius (Oscheius) andrassyi Tabassum and Shahina, 2008

Oscheius (Oscheius) carolinensis Ye, Torres-Barragán and Cardoza, 2010

= *Heterorhabditidoides (Oscheius) carolinensis* (Ye, Torres-Barragán and Cardoza, 2010) Zhang, Liu, Tan, Wang, Qiao, Yedid, Dai, Qiu, Yan, Tan, Su, Lai and Gao, 2012

Oscheius (Oscheius) caulleryi (Maupas, 1919) Sudhaus and Hooper, 1994

= *Rhabditis caulleryi* Maupas, 1919

= *Rhabditis (Rhabditis) caulleryi* Maupas, 1919

= *Rhabditis (Choriorhabditis) caulleryi* Maupas, 1919 (Osche, 1952)

= *Rhabditis (Oscheius) caulleryi* Maupas, 1919 (Sudhaus and Hooper, 1994)

Oscheius (Oscheius) chongmingensis (Zhang, Liu, Xu, Sun, Yang, An, Gao, Lin, Lai, He, Wu and Zhang, 2008) Ye, Torres-Barragán and Cardoza, 2010

= *Heterorhabditoides chongmingensis* Zhang, Liu, Xu, Sun, Yang, An, Gao, Lin, Lai, He, Wu and Zhang, 2008

= *Oscheius rugaoensis* (Zhang, Liu, Tan, Wang, Qiao, Yedid, Dai, Qiu, Yan, Tan, Su, Lai and Gao, 2012) Darsouei, Karimi and Shokoohi, 2014 n. syn.

= *Heterorhabditidoides rugaoensis* Zhang, Liu, Tan, Wang, Qiao, Yedid, Dai, Qiu, Yan, Tan, Su, Lai and Gao, 2012 n. syn.

= *Dolichorhabditis dolichura apud* Tabassum and Shahina (2002) *nec* Schneider (1866)

Oscheius (Oscheius) citri Tabassum, Shahina, Nasira and Erum, 2016

= *Oscheius cobbi* Tabassum, Shahina, Nasira and Erum, 2016

= *Oscheius cynodonti* Tabassum, Shahina, Nasira and Erum, 2016

= *Oscheius esculentus* Tabassum, Shahina, Nasira and Erum, 2016

= *Oscheius sacchari* Tabassum, Shahina, Nasira and Erum, 2016

= *Oscheius punctatus* Tabassum, Shahina, Nasira and Erum, 2016

= *Oscheius punctata* Tabassum, Shahina, Nasira and Erum, 2016 (lapsus)

Oscheius (Oscheius) colombianus Stock, Caicedo and Calatayud, 2005

= *Rhabditis (Oscheius) colombiana* Stock, Caicedo and Calatayud, 2005

= *Heterorhabditidoides (Oscheius) colombiana* (Stock, Caicedo and Calatayud, 2005) Zhang, Liu, Tan, Wang, Qiao, Yedid, Dai, Qiu, Yan, Tan, Su, Lai and Gao, 2012

Oscheius (Oscheius) esperancensis (Stock, 1990) Sudhaus, 2011

= *Rhabditis esperancensis* Stock, 1990

Oscheius (Oscheius) indicus Kumar, Jamal, Somvanshi, Chauhan and Mumtaz, 2019

Oscheius (Oscheius) lucianii (Maupas, 1919) Sudhaus and Hooper, 1994

= *Rhabditis lucianii* Maupas, 1919

= *Rhabditis (Choriorhabditis) lucianii* Maupas, 1919 (Osche, 1952)

= *Rhabditis (Oscheius) lucianii* Maupas, 1919 (Sudhaus and Hooper, 1994)

Oscheius (Oscheius) maqbooli Tabassum and Shahina, 2002

Oscheius (Oscheius) myriophilus (Poinar, 1986) Sudhaus and Hooper, 1994

= *Rhabditis myriophila* Poinar, 1986

= *Rhabditis (Rhabditis) myriophila* Poinar, 1986

= *Rhabditis (Oscheius) myriophila* Poinar, 1986 (Sudhaus and Hooper, 1994)

= *Heterorhabditidoides (Oscheius) myriophila* (Poinar, 1986) Zhang, Liu, Tan, Wang, Qiao, Yedid, Dai, Qiu, Yan, Tan, Su, Lai and Gao, 2012

= *Oscheius microvilli* Zhou, Yang, Wang, Bao, Wang, Hou, Lin, Yedig and Zhang, 2017 *in partim* (females while males belong to the genus *Caenorhabditis*) n. syn.

Oscheius (Oscheius) nadarajani Ali, Asif and Shaheen, 2011

Oscheius (Oscheius) necromenus (Sudhaus and Schulte, 1989) Sudhaus and Hooper, 1994

= *Rhabditis (Rhabditis) necromena* Sudhaus and Schulte, 1989

= *Rhabditis (Oscheius) necromena* Sudhaus and Schulte, 1989 (Sudhaus and Hooper, 1994)

Oscheius (Oscheius) rupaekramae (Khan, Singh and Kaushal, 2000) Sudhaus, 2011

= *Rhabditis rupaekramae* Khan, Singh and Kaushal, 2000

Oscheius (Oscheius) shamimi Tahseen and Nisa, 2006

Oscheius (Oscheius) siddiqii Tabassum and Shahina, 2010

= *Oscheius niazii* Tabassum and Shahina, 2010 n. syn.

Oscheius (Oscheius) wohlgemuthi (Völk, 1950) Tahseen and Nisa, 2006

= *Rhabditis wohlgemuthi* Völk, 1950

= *Rhabditis (Choriorhabditis) wohlgemuthi* Völk, 1950 (Osche, 1952)

= *Rhabditis aspera apud* Örley (1886), *nec* Bütschli (1873)

Species inquirendae

Oscheius (Oscheius) basothovii Lephoto and Gray, 2019

Oscheius (Oscheius) safricanus Serepa-Dlamini and Gray, 2018

= *Oscheius safricana* Serepa-Dlamini and Gray, 2018 (lapsus)

Subgenus *Dolichorhabditis* Andrássy, 1983

syn. *Dolichorhabditis* Andrássy 1983

Diagnosis

Stoma tubular or barrel-shaped with metastegostom bearing setose teeth. Bursa peloderan. Male tail tip not reaching beyond bursa end. Spicule tips shaped like a probe head.

Type species

Oscheius (Dolichorhabditis) dolichura (Schneider, 1866) Sudhaus and Hooper, 1994

= *Leptodera dolichura* Schneider, 1866

= *Rhabditis dolichura* (Schneider, 1866) Bütschli, 1873

= *Rhabditis (Caenorhabditis) dolichura* (Schneider, 1866) Bütschli, 1873 (Osche, 1952)

= *Caenorhabditis dolichura* (Schneider, 1866) Osche, 1952 (rank by Dougherty, 1955)

= *Rhabditis (Pellioditis) dolichura* (Schneider, 1866) Bütschli, 1873 (Sudhaus, 1976)

= *Rhabditis (Oscheius) dolichura* (Schneider, 1866) Bütschli, 1873 (Sudhaus and Hooper, 1994)

= *Dolichorhabditis dolichura* (Schneider, 1866) Andrássy, 1983

= *Rhabditis herfsi* Rahm, 1924

Other species

Oscheius (Dolichorhabditis) bengalensis (Timm, 1956) Sudhaus and Hooper, 1994

= *Rhabditis (Choriorhabditis) marina bengalensis* Timm, 1956

= *Pellioditis marina bengalensis* (Timm, 1956) Timm, 1960

= *Rhabditis bengalensis* Timm, 1956

= *Rhabditis (Pellioditis) bengalensis* Timm, 1956 (Sudhaus, 1974)

= *Rhabditis (Oscheius) bengalensis* Timm, 1956 (Sudhaus and Hooper, 1994)

= *Oscheius bengalensis* Timm, 1956 (Sudhaus and Hooper, 1994)

= *Dolichorhabditis bengalensis* (Timm, 1956) Andrássy, 2005

= *Rhabditis bengalensis mexicana* Hopper, 1963

Oscheius (Dolichorhabditis) karachiensis (Mehmood and Khanum, 2018) Abolafia and Peña-Santiago, 2019

= *Oscheius karachiensis* Mehmood and Khanum, 2018

Oscheius (Dolichorhabditis) debilicauda (Fuchs, 1937) Abolafia and Peña-Santiago, 2019

= *Rhabditis debilicauda* Fuchs, 1937

= *Rhabditis (Caenorhabditis) debilicauda* Fuchs, 1937 (Osche, 1952)

= *Caenorhabditis debilicauda* (Fuchs, 1937) Osche, 1952

= *Dolichorhabditis debilicauda* (Fuchs, 1937) Andrássy, 1983

Oscheius (Dolichorhabditis) dolichuroides (Anderson and Sudhaus, 1985) Sudhaus and Hooper, 1994

= *Rhabditis (Pellioditis) dolichuroides* Anderson and Sudhaus, 1985

= *Rhabditis (Oscheius) dolichuroides* Anderson and Sudhaus, 1985 (Sudhaus and Hooper, 1994)

= *Oscheius dolichuroides* Anderson and Sudhaus, 1985 (Sudhaus and Hooper, 1994)

= *Dolichorhabditis dolichuroides* (Anderson and Sudhaus, 1985) Andrássy, 2005

Oscheius (Dolichorhabditis) dux (Gorgadze, 2010) Sudhaus, 2011

= *Dolichorhabditis dux* Gorgadze, 2010

= *Oscheius dux* (Gorgadze, 2010) Sudhaus, 2011

Oscheius (Dolichorhabditis) guentheri (Sudhaus and Hooper, 1994) Andrássy, 2005

= *Dolichorhabditis guentheri* (Sudhaus and Hooper, 1994) Andrássy, 2005

= *Oscheius guentheri* (Sudhaus and Hooper, 1994) Andrássy, 2005

Oscheius (Dolichorhabditis) janeti (Lacaze-Duthiers in Janet, 1893) Sudhaus, 2011

= *Pelodera janeti* De Lacaze-Duthiers in Janet, 1893 (*nomen nudum*)

= *Rhabditis janeti* (De Lacaze-Duthiers in Janet, 1893) de Man, 1894

= *Rhabditis (Protorhabditis) janeti* (De Lacaze-Duthiers in Janet, 1893) de Man, 1894 (Osche, 1952)

= *Oscheius janeti* (Lacaze-Duthiers in Janet, 1893) Sudhaus, 2011

Oscheius (Dolichorhabditis) latus (Cobb, 1906) Sudhaus, 2011

= *Rhabditis latus* Cobb, 1906

= *Oscheius latus* (Cobb, 1906) Sudhaus, 2011

Oscheius (Dolichorhabditis) onirici Torrini, Mazza, Carletti, Benvenuti, Roversi, Fanelli, de Luca, Troccoli and Tarasco, 2015

= *Oscheius onirici* Torrini, Mazza, Carletti, Benvenuti, Roversi, Fanelli, de Luca, Troccoli and Tarasco, 2015

= *Oscheius tipulae* apud Abolafia and Lechuga-Puñal (2014), nec Lam and Webster (1971)

Oscheius (Dolichorhabditis) pseudodolichura (Körner in Osche, 1952) Sudhaus and Hooper, 1994

= *Rhabditis (Caenorhabditis) pseudodolichura* Körner in Osche, 1952

= *Caenorhabditis pseudodolichura* Körner in Osche, 1952

= *Pellioditis pseudololichura* (Körner in Osche, 1952) Andrássy, 1983

= *Rhabditis (Oscheius) pseudodolichura* Körner in Osche, 1952 (Sudhaus and Hooper, 1994)

= *Oscheius pseudodolichura* Körner in Osche, 1952 (Sudhaus and Hooper, 1994)

= *Rhabditis (Choriorhabditis) pseudodolichura* Körner in Osche, 1952 (Mengert, 1953)

= *Dolichorhabditis pseudodolichura* (Körner in Osche, 1952) Andrássy, 2005

Oscheius (Dolichorhabditis) saproxylicus Abolafia and Peña-Santiago, 2019

Oscheius (Dolichorhabditis) sechellensis (Potts, 1910) Sudhaus and Hooper, 1994

= *Rhabditis sechellensis* Potts, 1910

= *Rhabditis (Choriorhabditis) sechellensis* Potts, 1910 (Osche, 1952)

= *Rhabditis (Pellioditis) sechellensis* Potts, 1910 (Sudhaus, 1976)

= *Rhabditis (Oscheius) sechellensis* Potts, 1910 (Sudhaus and Hooper, 1994)

= *Oscheius sechellensis* (Potts, 1910) Sudhaus and Hooper, 1994

= *Dolichorhabditis sechellensis* (Potts, 1910) Andrássy, 2005

= *Oscheius sechellensis* (Potts, 1910) Sudhaus and Hooper, 1994

Oscheius (Dolichorhabditis) tereticorpus (Kito and Ohyama, 2008) Sudhaus, 2011

= *Dolichorhabditis tereticorpus* Kito and Ohyama, 2008

= *Oscheius tereticorpus* (Kito and Ohyama, 2008) Sudhaus, 2011

Oscheius (Dolichorhabditis) tipulae Lam and Webster, 1971

= *Rhabditis (Rhabditella) tipulae* Lam and Webster, 1971

= *Rhabditis (Oscheius) tipulae* Lam and Webster, 1971 (Sudhaus, 1993)

= *Oscheius tipulae* Lam and Webster, 1971 (rank by Sudhaus, 2011)

= *Dolichorhabditis tipulae* (Lam and Webster, 1971) Andrássy, 2005

Oscheius (Dolichorhabditis) zarinae (Khan, Singh and Kaushal, 2000) Sudhaus, 2011

= *Rhabditis zarinae* Khan, Singh and Kaushal, 2000

= *Oscheius zarinae* (Khan, Singh and Kaushal, 2000) Sudhaus, 2011

Species inquirendae

Oscheius (Dolichorhabditis) oxyuris (Claus, 1862) Abolafia and Peña-Santiago, 2019
= *Anguillula oxyuris* Claus, 1862, nec *Rhabditis oxyuris* apud Bütschli (1873)
