# Supplementary material for: Morphological, Morphometrical and Molecular Characterization of Oscheius siddiqii Tabassum and Shahina, 2010 (Rhabditida, Rhabditidae) from India with Its Taxonomic Consequences for the Subgenus Oscheius Andrássy, 1976
Source: Biology (Basel). 2021 Nov 27;10(12):1239. doi: 10.3390/biology10121239 (PMC8698764; doi:10.3390/biology10121239)
Supplement: Supplementary file 1 [file biology-10-01239-s001.zip › Supplementary table S2.pdf]

Table S2. Comparative morphometrics of the females of the species of the subgenus *Oscheius*. All measurements in μm except indexes.

Species	Reference	Country	L	*a*	*b*	*c*	*c′*	V	Lip width	Stoma length	Isthmus length	Bulb length	NR– ant. End	EP– ant. end	EP%	EP position	Pharynx length	MBD	ABD	Rectum length	Rectum / ABD	Tail
andrassyi	Tabassum & Shahina (2008)	Pakistan	1322–1962	15–21	6.7–9.4	11–18	3.0–4.3	50–53	8*	14–18	35–50	30–40	130–165	160–225	109*	Bulb	175–215	70–122	25–40	75–120	3.0–4.0	100–130
basothovii	Lephoto & Gray (2019)	South Africa	2056–2210	11*	10.9*	22*	1.6*	64–6	13*	10–12	85–87	68–69	?	51–52	73*	Bulb	173–174	178–180	56–60	91–92	1.6*	90–92
carolinensis	Ye et al. (2010)	USA	1360–2420	15–23	5.4–9.5	8–18	3.2–6.1	47–55	13*	18–27	38–65	35–62	148*	205–262	84*	Isthmus	228–264	67–123	28–43	69*	2.0–3.0	108–206
caulleryi	Maupas (1919)	Algeria	1910–2364	?	9.1–9.7	20–25	2.5	?	?	16–20	?	?	?	?	?	?	210–244	111–122	?	30–35	1.0	96
chongmingensis	Zhang et al. (2008)	China	1640–2220	18*	5.2*	12*	2.5*	50–54	6*	10–12	38*	24*	102–156	127–180	105*	Bulb to intestine	154–202	44–67	20–27	41	1.4	67–102
chongmingensis	Liu et al. (2012)	Mongolia	1313–2182	14–21	5.6–9.0	8–13	3.1–4.8	44–52	9*	14–19	36*	35–47	133–223	145–266	90	Bulb	188–297	74–141	31–43	43–69	1.4–1.6	121–188
citri and its synonyms	Tabassum et al. (2016)	Pakistan	1201–1814	15–21	6.7–9.4	10–17	3.4–5.4	45–55	12–15	14–19	35–46	31–34	121–187	148–222	98*	Bulb to intestine	172–225	65–112	20–33	40–56	1.8–2.0	88–144
citri	Rana et al. (in revision)	India	1370–2348	14–23	6.4–11.0	12–24	2.7–4.2	48–52	6–11	14–21	21–39	33–53	130–201	144–223	79–105	Bulb to intestine	167–224	66–146	28–40	34–86	1.2–2.2	87–151
colombianus	Stock et al. (2005)	USA	923–1805	15–19	5.2–8.0	8–10	3.5–5.0	47–57	7–9	21–28	39–48	26–41	124–174	157–165	82*	Bulb	176–225	49–106	22–38	47*	1.5*	51–71
esperancensis	Stock (1990)	Argentina	1273–1800	15–22	5.9–7.5	9–17	5.0*	45–52	9*	14–20	46*	29*	112–200	134–212	85*	Bulb	192–260	70–106	21–44	40*	1.9*	104–160
indicus	Kumar et al. (2019)	India	1072–1480	17–21	5.7–7.1	8–10	5.0–6.6	45–50	11–15	13–18	37–47	33–41	118–162	154–201	91*	Bulb to intestine	181–210	58–82	23–28	58–76	2.6–2.9	131–156
lucianii	Maupas (1919)	Algeria	2100–2817	?	6.2–10.0	15–18	?	?	10*	10–18	?	?	?	?	?	Bulb to intestine	243–276	85–143	?	?	?	90–185
lucianii	Chitwood (1933)	USA	1360–1450	?	?	?	?	?	?	20–26	54–80	30–50	160–210	210–250	?	Bulb to intestine	130–170	114–120	?	?	?	82–130
myriophilus	Poinar (1986)	California	1200–1500	19–21	6.8–7.7	11–13	4.2–5.3	49–51	9–13	18–21	44*	35*	126–146	165–190	95*	Bulb	174–193	57–70	22–28	56–96	2.2–3.5	108–135
myriophilus as *microvilli*	Zhou et al. (2017)	China	864–1446	23–36	4.4–6.2	6–23	7.3*	47–66	6*	14–22	48*	36*	144*	164–222	92*	Bulb	177–282	23–50	9–25	37*	1.9*	89–194
myriophilus as *safricanus*	Dlamini & Gray (2018)	South Africa	894–1413	8–15	4.8–10.0	14–16	2.2*	28–58	6*	7–9	34–40	28*	141–179	120–250	84*	Isthmus base	145–213	59–150	25–45	55*	1.4*	50–89
maqbooli	Tabassum & Shahina (2002)	Pakistan	942–1342	14–18	4.8–5.6	7–11	4.6–5.9	49–53	13–16	19*	58*	32–40	128–164	150–200	82*	Isthmus	182–237	53–92	20–29	61–118	3.0–4.0	112–148
nadarajani	Ali et al. (2011)	India	1358–1606	18–20	5.0–5.2	11–13	5.1–5.2	52.5*	10*	18–19	27*	33*	165–201	175–194	76*	Isthmus	256–267	78–79	23–26	42–63	1.8–2.4	121–123

necromenus	Sudhaus & Schulte (1989)	Australia	830–1500	15–20	4.2–6.3	10–14	3.7–2.9	41–56	16–17	13–21	56*	27–38	165*	157–216	90*	Bulb	189–239	54–90	22–45	59–99	2.2–2.7	81–131
necromenus	Valizadeh et al. (2017)	Iran	958–1580	14–25	5.1–8.5	8–16	2.4–7.4	41–52	10*	13–18	?	25*	?	?	?	Bulb	143–193	49–116	16–42	?	?	91–127
necromenus	Carta et al. (2018)	Australia	1340–2120	9–15	6.1–8.8	12–17	2.3–2.9	47–54	?	21–25	?	?	?	?	?	Bulb	?	?	?	?	?	?
rugaoensis	Zhang et al. (2012)	China	1639–2259	19–28	6.0–9.4	12.6–13	4.3*	48–54	10*	18*	45*	25*	135*	222–278	92*	Bulb	237–304	71-106	26–39	50*	1.5*	113–155
rugaoensis	Darsouei et al. (2014)	Iran	1562–1600	8–9	8.0–9.0	15–22	2.2–3.0	47–48	11–14	19–24	39–44	32–41	160–168	197–229	88*	Bulb	176–193	93–119	31–34	80–88	2.6*	71–105
rugaoensis	Carta et al. (2018)	Australia	1340–2120	9–15	6.0–9.0	12–17	2.3–2.9	47–54	?	21–25	?	?	?	?	?	Bulb	?	100–180	?	?	?	?
shamimi	Tahseen & Nisha (2006)	India	1360–2420	12–20	4.2–6.3	7–14	4.1–5.8	45–51	11–14	19–23	58*	26–30	112–165	137–190	74*	Isthmus to bulb	181–241	58–97	21–32	56–85	2.2–2.6	110–132
shamimi	Tabassum & Shahina (2010)	Pakistan	1076–1422	17–19	6.0–8.0	9–11	4.0–5.2	45–49	?	16–18	36–52	33–37	130–160	150–180	?	Isthmus to bulb	178–208	60–80	24–30	32*	1.2*	108–140
siddiqii	Tabassum & Shahina (2010)	Pakistan	1130–1390	15–18	6.0–6.9	10–13	3.5–5.4	40–52	10*	13–17	45–50	30–38	130–160	155–197	93*	Bulb	185–204	73–90	24–31	48–93	2.0–3.0	100–132
siddiqii as *niazii*	Tabassum & Shahina (2010)	Pakistan	837–1487	15–17	4.4–7.8	11–14	2.8–4.0	48–54	6*	15–25	40–45	35–40	140–170	130–200	97*	Bulb	180–200	50–100	20–35	50–85	2.5–3.4	75–175
siddiqii	Present study	India	1121–1697	14–19	5.6–8.9	6–15	3.5–7.5	45–61	6–8	13–20	45–53	26–34	136–181	172–220	88–113	Bulb to intestine	169–196	72–106	22–38	59–78	2.0–3.2	123–180
wohlgemuthi as *R. aspera*	Örley (1880, 1886a,b)	Hungary	2000	28	9.0	13	?	?	?	?	?	?	?	?	?	Bulb	222	71	?	?	?	154
wohlgemuthi	Völk (1950)	Germany	1570–2100	16–19	6.4–7.6	10–14	6.0–7.0	48–53	8*	24	27*	22*	?	?	?	Bulb	270–284	40–69	18–25	28*	1.5*	108–176
wohlgemuthi	Abolafia & Santiago (2010)	Spain	1639–2259	19–28	6.0–9.4	13	6.1*	48–54	8*	15*	32*	26*	134*	144*	93*	Bulb	237–304	71-106	26–39	41*	1.4*	180–216

*Measurements obtained from drawings; ? = measurements unknown.
