# Supplementary material for: Morphological, Morphometrical and Molecular Characterization of Oscheius siddiqii Tabassum and Shahina, 2010 (Rhabditida, Rhabditidae) from India with Its Taxonomic Consequences for the Subgenus Oscheius Andrássy, 1976
Source: Biology (Basel). 2021 Nov 27;10(12):1239. doi: 10.3390/biology10121239 (PMC8698764; doi:10.3390/biology10121239)
Supplement: Supplementary file 1 [file biology-10-01239-s001.zip › Supplementary table S3.pdf]

Table S3. Comparative morphometrics of the males of the species of the subgenus *Oscheius*. All measurements in µm except indexes.

Species	Reference	Country	L	*a*	*b*	*c*	*c′*	Lip width	Stoma length	Isthmus length	Bulb length	NR–ant. End	EP–ant. End	EP%	EP position	Pharynx length	MBD	ABD	Tail length	SL	GL	GS (GL/SL)
andrassyi	Tabassum &Shahina (2008)	Pakistan	1025–1032	20–25	6.2–7.0	18–25	1.1–2.1	?	15–17	28–40	25–30	123–160	167–198	102*	Bulb	158–178	45–51	30–40	45–63	45–51	20–25	0.4–0.5
basothovii	Lephoto & Gray (2019)	South Africa	889–1454	10*	6.5*	17*	1.3*	?	?	74–78	50–60	?	39–40	?	Isthmus	152–157	107–109	45–48	55–63	47–49	?	?
carolinensis	Ye et al. (2010)	USA	1000–2000	16–24	4.9–8.9	19–50	1.0–2.3	6*	18–23	33–58	32–52	?	177–295	?	Bulb	165–245	52–89	25–40	32–64	50–81	20–35	0.3–0.4
caulleryi	Maupas (1919)	Algeria	1054–1310	?	5.9–6.3	23–30	?	?	?	?	?	?	?	?	?	?	?	?	?	39–48	?	?
chongmingensis	Zhang et al. (2008)	China	822–1400	22*	7.2*	37*	1.0*	5*	15*	38*	27*	88–133	124–193	102*	Bulb to intestine	113–186	38–62	21–33	22–39	37–68	20–33	0.4–0.5
chongmingensis	Liu et al. (2012)	Mongolia	848–1414	17–25	4.3–6.6	18–29	1.2–1.8	?	13–16	?	27–43	137–172	156–215	92*	Bulb	184–235	43–74	25–38	38–56	40–59	19–32	0.5*
citri and its synonyms	Tabassum et al. (2016)	Pakistan	864–1335	17–27	5.4–7.3	22–36	1.1–1.9	10–13	14–18	32*	40*	118–166	138–190	87*	Bulb to intestine	155–196	44–66	23–31	28–46	57–67	21–27	3.5–4.7
citri	Rana et al. (in revision)	India	1024–1433	16–22	5.3–8.1	25–41	1.2–2.1	5–8	11–18	17–33	33–46	96–150	149–211	71–104	Bulb	154–199	54–73	20–31	32–51	42–75	21–31	0.3–0.5
colombianus	Stock et al. (2005)	USA	665–1163	16–29	3.9–5.4	13–16	2.1–3.5	7–9	19–24	25–59	25–40	109–155	116–158	?	Bulb	168–213	23–72	15–32	51–70	43–68	16–24	0.3–0.4
esperancensis	Stock et al. (1990)	Argentina	1125–1210	16–20	5.8–7.0	16–21	?	?	15–17	36*	25*	135–153	154–160	86*	Bulb	186–212	65–83	39*	18–22	35–42	8–12	0.2
indicus	Kumar et al. (2019)	India	886–1209	17–22	4.9–6.9	26–36	1.2–1.4	11–13	14–17	31–41	27–32	122–146	158–187	101*	Bulb to intestine	159–181	46–64	24–28	32–39	54–66	24–28	0.3–0.4
lucianii	Maupas (1919)	Algeria	1215–1900	?	?	?	?	?	12–18	?	?	?	?	?	Bulb to intestine	168–271	57–78	?	42	60	?	?
lucianii	Chitwood (1933)	USA	1000–1320	?	?	?	?	?	20–22	50–54	24–35	156–180	180–230	?	Bulb to intestine	126–140	64–94	44–60	?	50–62	20–27	?
maqbooli	Tabassum & Shahina (2002)	Pakistan	720–1165	13–19	3.9–5.3	19–27	1.3–1.6	12–16	10–18	50*	32–40	124–176	144–177	80*	Isthmus	184–248	40–69	28–36	32–48	48–60	19–32	0.4–0.5
myriophilus	Poinar (1986)	California	830–1470	18–22	5.2–7.4	15–20	1.7*	9–13	16–19	?	?	117–165	149–229	97*	Bulb	161–200	38–80	32–45	56–72	32–54	19–32	0.5–0.6
myriophilus as *microvilli*	Zhou et al. (2017)	China	639–939	24–29	3.4–4.7	13–20	6.2*	5*	13–22	24*	22*	151*	157–217	94*	Bulb	162–214	20–40	15–28	34–55	25–42	16–33	0.6–1.1
myriophilus as *safricanus*	Dlamini & Gray (2018)	South Africa	864–1309	11–17	3.4–10.0	21–25	1.4*	?	?	34	?	86–145	111–256	?	Isthmus base	108–198	48–93	22–35	32–58	28–60	21–32	0.5–0.8
nadarajani	Ali et al. (2011)	India	1191–1395	23–28	4.5–5.3	34–38	1.3–1.8	9*	19	25*	30*	160–168	166–175	71*	Isthmus	242–256	41–47	23–29	32–36	58–63	40*	0.6
necromenus	Sudhaus and Schulte (1989)	Australia	670–950	14–19	4.3–5.0	12–18	1.7*	16–17	13–21	?	23–27	?	121–166	?	Bulb	158–194	38–50	32–66	54–63	34–44	12–23	0.4–0.5
rugaoensis	Zhang et al. (2012)	China	1195–1692	16–26	3.8–6.4	7–11	1.8*	6*	18*	33*	22*	?	202–412	93*	Bulb	219–444	46–66	17–53	50*	35–61	10–26	0.2–0.5

rugaoensis	Darsouei et al. (2014)	Iran	1082–1100	16–18	2.9–6.6	28–30	1.1–1.2	13–14	17–22	36–37	29–32	141–161	180–205	92*	Isthmus	163–361	60–70	28–33	36–38	58–66	28–30	0.5*
siddiqii	Tabassum & Shahina (2010)	Pakistan	828–998	18–21	4.3–5.6	20–24	1.3–1.5	10*	14–15	40–52	28–37	120–140	150–172	?	Bulb	165–180	43–53	28–30	38–45	50–55	20–25	0.4–0.5
siddiqii* as *niazii	Tabassum & Shahina (2010)	Pakistan	715–1020	16–17	4.4–5.8	18–26	1.0–1.8	?	?	30–50	30–40	130–165	130–160	?	Bulb	160–175	45–57	20–37	35–40	42–52	18–30	0.4–0.6
siddiqii	Present study	India	916–1441	15–27	5.1–7.7	20–33	1.4–2.5	5–8	13–20	35–49	26–67	125–159	162–220	94–124	Bulb to intestine	148–176	38–61	20–27	36–49	39–55	21–26	0.4–0.5
shamimi	Tahseen & Nisha (2006)	India	938–1118	17–20	5.1–5.5	25–31	1.1–1.5	12–14	18–22	46*	31*	124–148	166–192	72*	Isthmus	181–203	49–57	25–31	29–38	53–67	22–28	0.3–0.4
shamimi	Tabassum & Shahina (2010)	Pakistan	822–1052	19–25	5.0–7.0	20–30	1.1–1.6	?	14–16	32–42	30–32	100–135	140–175	?	Isthmus to bulb	160–168	40–50	25–31	35–45	50–57	20–25	0.4–0.44
wohlgemuthi as *R. aspera*	Örley (1880, 1886a,b)	Hungary	2000	28	9.0	24	?	?	?	?	?	?	?	?	Bulb	222	36	?	83	?	?	?
wohlgemuthi	Völk (1950)	Germany	1170–1520	18–20	5.6–7.3	22–30	?	?	19	?	?	?	?	?	?	200–235	59	46–57	34	54–66	16	0.3
wohlgemuthi	Abolafia & Santiago (2010)	Spain	1130	19	7.3	28	1.2	?	12	19	39	32	118	?	Bulb	156	59	41	34	?	?	?

*Measurements obtained from drawings; ? = measurements unknown.
